# Decoupling Solvent Effects: Perfluorinated Sulfonic Acid Structure and Catalyst Layer Stability in PEM Water Electrolysis

**DOI:** 10.3390/membranes16010022

**Published:** 2026-01-01

**Authors:** Jong-Hyeok Park, Jin-Soo Park

**Affiliations:** 1 Department of Civil, Environmental, and Biomedical Engineering, The Graduate School, Sangmyung University, Cheonan 31066, Republic of Korea; parkjh8254@kims.re.kr; 2 Future Environment and Energy Research Institute, Sangmyung University, Cheonan 31066, Republic of Korea; 3 Department of Green Chemical Engineering, College of Engineering, Sangmyung University, Cheonan 31066, Republic of Korea

**Keywords:** proton exchange membrane water electrolysis, catalyst layer, perfluorosulfonic acid ionomer, side-chain chemistry, ionomer thin-film formation

## Abstract

Solvent differences in perfluorinated sulfonic acid ionomer (PFSI) dispersions confound comparisons of catalyst-layer (CL) binders for PEM water electrolysis. We use a propylene-glycol (PG) single-solvent platform to isolate effects of ionomer structure, i.e., side-chain architecture and equivalent weight (EW), across five PFSIs (Nafion D2021, Nafion D2020, Aquivion D98-25BS, Aquivion D72-25BS, and 3M E-22397B) as hydrogen evolution reaction-CL binders. PG collapses ionomer aggregates from microns to sub-100 nm and reduces catalyst–ionomer agglomerates without a systematic EW trend, indicating that performance differences originate during film formation. PG makes CLs more hydrophobic (higher *θ_R_*), yet aerophobicity (*θ_Air_*) still increases with decreasing EW, consistent with higher sulfonic-acid density. Under PG, current density at 1.9 V rises with decreasing EW, with thicker short-side chain/mid-side chain (SSC/MSC) films (≥140 nm) and lower ohmic overpotentials than long-side-chain (LSC) Nafion. Durability under Accelerated Stress Test-2 (AST-2; 0.3/3.0 A cm^−2^, 48 h) splits by class: LSC Nafion shows the largest degradation slopes, whereas SSC/MSC are lower; transmission electron microscope images show Pt growth with Nafion D2020-PG but minimal growth with Aquivion D98-25BS-PG. With solvent effects removed, ionomer structure governs CL performance and stability; SSC (EW ≈ 980) is recommended, and MSC maximizes current with moderate stability.

## 1. Introduction

The catalyst layer (CL), which largely governs the performance and durability of proton exchange membrane water electrolysis (PEMWE), is a porous composite of electrocatalyst particles and a perfluorosulfonic acid ionomer (PFSI) binder. Because the CL is the reaction zone where proton conduction, water management, and the oxygen evolution charge-transfer process occur, changes in CL microstructure and ionomer properties have a far greater impact on performance than membrane or bulk transport losses. Therefore, rational tuning of the CL is essential for achieving high efficiency and long-term stability in PEMWE. Within this microstructured layer, the PFSI (i) provides proton-conduction pathways, (ii) mechanically binds catalyst agglomerates, and (iii) helps maintain proper hydration; hence, rational ionomer selection is critical for achieving both high efficiency and long-term stability [1,2,3,4,5,6].

The physicochemical structure of a PFSI can be described by three main parameters: (i) the side-chain chemistry, (ii) the equivalent weight (EW), and (iii) the dispersing solvent environment. Side-chain chemistry determines the acidity of functional groups and the hydrophilic/hydrophobic balance of the ionomer, thereby controlling water uptake, swelling behavior, and interfacial interactions with catalyst particles. Equivalent weight (EW) dictates the density of sulfonic acid groups in the polymer, which directly affects proton conductivity and ionic domain connectivity, as well as mechanical integrity of the ionomer network. The solvent environment influences ionomer solvation, aggregation state, and dispersion behavior in the ink, which ultimately governs CL morphology, porosity, and triple-phase boundary formation during drying and film formation [7,8,9]. Nonetheless, much of the prior work has optimized ionomer content (ionomer-to-carbon, I/C) and examined structural factors (side-chain length and EW) with respect to CL morphology, transport, and electrochemical response [2,10,11,12,13,14,15,16]. These studies collectively show that ionomer content reshapes porosity, water uptake, and resistances in the CL, while side-chain architecture and EW modulate thin-film morphology, wettability, and proton conductivity, thereby altering device performance [17,18,19,20,21,22,23,24]. Recent reports further emphasize that the dispersion state of the ionomer, influenced by side-chain length and EW, controls film formation around catalyst particles, triple-phase boundary (TPB) density, and local hydration in both PEMFC and PEMWE environments [25,26].

However, commercial ionomer dispersions differ in their as-received solvent systems (e.g., water–alcohol mixtures for Nafion and 3M vs. water-based Aquivion), and additional polar protic organic solvents are typically introduced during ink formulation. As a result, solvent effects can be convolved with ionomer-structure effects, obscuring the intrinsic role of ionomer chemistry. Our recent study addressed the solvent dimension by fixing the ionomer (Aquivion D98-25BS) and systematically varying glycol-based dispersing solvents. Solvents with more negative solvation energies (e.g., EG, PG) improved ionomer dispersion, resulting in more homogeneous CLs and higher initial current densities. However, their effects on durability differed: while EG showed stronger performance gains, it also exhibited faster degradation, whereas PG provided both stable dispersion and slower performance decay. For this reason, PG offered the clearest balance between performance and long-term durability [8]. Building on this, propylene glycol (PG) is adopted here as a standardized single-solvent medium to decouple and interrogate the intrinsic effects of ionomer structure, such as side-chain chemistry and EW, under a controlled dispersion environment.

Accordingly, this work investigates how ionomer side-chain architecture and EW govern CL microstructure, transport resistances, and electrochemical durability in PEMWE under a single-solvent (PG) system. Five commercially available PFSIs, Nafion D2021 and D2020 (long side-chain, LSC), Aquivion D98-25BS and D72-25BS (short side-chain, SSC), and 3M E-22397B (mid-side-chain, MSC), are individually dispersed in PG and applied to fabricate CLs. By integrating ink/CL characterization with polarization and accelerated durability testing, this study aims to elucidate structure–property–performance relationships of PFSI binders independent of solvent variability. Furthermore, by establishing a solvent-replacement approach applicable to CL fabrication and deepening the understanding of ionomer–solvent interactions relevant to oxygen-electrode formation, this work provides practical insights for designing and optimizing electrode architectures that enhance both PEMWE performance and operational longevity.

## 2. Experiment

### 2.1. Fabrication and Characterization of Single-Solvent Ionomer Dispersions

**Figure 1:**
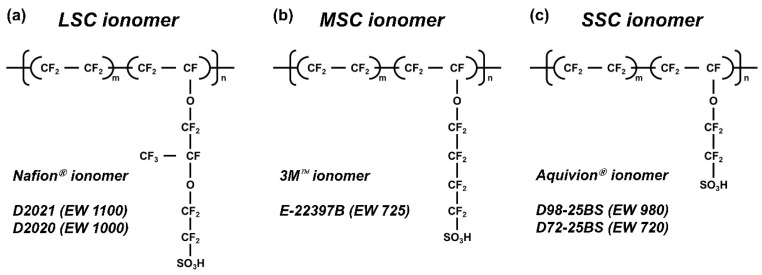
Chemical structures and classification of PFSI used in this study, grouped by side-chain length (LSC, MSC, SSC) and labeled with EW.

Commercial PFSI dispersions were used as received: Nafion D2021 and Nafion D2020 (EW 1100 and 1000; 20 wt%; Chemours, Wilmington, DE, USA), Aquivion D98-25BS and Aquivion D72-25BS (EW 980 and 720; 25 wt%; Solvay, Brussels, Belgium), and 3M E-22397B (EW 725; 20 wt%; 3M, Saint Paul, MN, USA). Propylene glycol (PG; JUNSEI Chemical Co., Ltd., Tokyo, Japan) served as the solvent for solvent substitution. Basic ionomer properties, i.e., EW, number of tetrafluoroethylene (TFE) repeat units, and side-chain structure, are summarized in Figure 1 and Table 1.

PG-based ionomer dispersions with a solids content of 10 wt% were prepared using a rotary evaporator (RE100-pro, DLAB Scientific Co., Ltd., Beijing, China). Briefly, 18 g of PG and each commercial ionomer dispersion, specifically 10 g of Nafion, 8 g of Aquivion, and 10 g of 3M, were combined in a flask, and water or water/alcohol (W/A) was selectively removed by rotary evaporation based on boiling-point differences at 90 °C, 30 rpm, and −0.3 bar, while retaining PG. The resulting solvent-substituted dispersions are 20 g of each Nafion D2021-PG, Nafion D2020-PG, Aquivion D98-25BS-PG, Aquivion D72-25BS-PG, and 3M E-22397B-PG. Consequently, to decouple solvent-related influences, propylene glycol (PG) was employed as a standardized single-solvent medium in order to evaluate the intrinsic effects of ionomer structure under a controlled and uniform dispersion environment.

Thermogravimetric analysis (TGA; TG209 F1 Libra, NETZSCH, Selb, Germany) was performed to quantify solids content and identify solvent evaporation temperatures. Measurements were conducted under a nitrogen atmosphere from ambient temperature to 500 °C at 10 °C min^−1^ [27,28]. Based on the boiling points of the respective solvents, both the as-received and PG-substituted ionomer dispersions were analyzed to confirm selective solvent removal and the resulting solids content. The particle size of ionomer aggregates in the as-received and PG-substituted dispersions was measured by dynamic light scattering (DLS; ELSZ-1000, Otsuka, Osaka, Japan), which was measured five times [29,30]. Prior to DLS, each dispersion was diluted to 0.01 wt% using the same dispersing solvent present in that dispersion (i.e., PG for PG-based dispersions and water or W/A for as-received dispersions) [25,31].

### 2.2. Fabrication and Characterization of Catalyst Ink, CL, and Membrane Electrode Assembly

Hydrogen evolution reaction (HER) inks were prepared by mixing a Pt/C electrocatalyst (TKK TEC10E50E, 47 wt% Pt; TANAKA, Tokyo, Japan) with an ionomer dispersion and, when required, a small amount of additional solvent to adjust viscosity. Both as-received and PG-substituted ionomer dispersions were used. The inks were formulated at an ionomer-to-carbon (I/C) ratio of 0.9 and a total solids content of 8.0 wt%. Inks were coated onto a PTFE-coated polyimide release film to target a Pt loading of 0.8 mg cm^−2^. To ensure complete removal of propylene glycol (PG) from the HER-CLs, the coated electrodes were dried at 120 °C for 24 h under vacuum. This drying condition was selected based on thermogravimetric analysis (TGA), which indicates that PG evaporation initiates at approximately 120 °C. Under these conditions, complete solvent removal is achieved. The dried HER-CL films were cut into 4 cm^2^ pieces and transferred onto one side of a Nafion 212 membrane (50 µm; Chemours, Wilmington, DE, USA) at 10 MPa and 120 °C for 3 min. Notably, insufficient drying results in unsuccessful decal transfer, providing an additional practical verification of complete solvent evaporation.

Oxygen evolution reaction (OER) inks were prepared from iridium oxide (IrO_2_, ≥84.5% Ir; Alfa Aesar, Haverhill, MA, USA), Nafion D521 ionomer dispersion (5 wt% in alcohol; Chemours), deionized water, and 2-propanol (Carlo Erba Reagents, Cornaredo, Italy). The OER ink contained 20 wt% ionomer and 3.5 wt% solids. The OER CL was directly coated onto Nafion 212 to an IrO_2_ loading of 1.0 mg cm^−2^ over a 4 cm^2^ active area [32].

The particle-size distribution of catalyst-ionomer agglomerates in the inks was measured by dynamic light scattering (DLS). Inks prepared under the same conditions as for CL fabrication were diluted to 0.1 wt% using the same solvent system as the ink to probe catalyst-ionomer interactions in a reproducible method.

Contact angles of CL surfaces were measured with a contact-angle goniometer (Theta Lite; Biolin Scientific, Stockholm, Sweden) to assess wettability (water in air) and to calculate aerophobicity (bubble in water), which was measured five times. The aerophobic contact angle (*θ_bubble in water_*, here denoted *θ_Air_*) was obtained from the water-in-air contact angle (*θ_water in air_*, here denoted *θ_R_*) using:

*θ_Air_* = 180° − *θ_R_*

A larger *θ_Air_* (i.e., smaller *θ_R_*) indicates a more aerophobic surface, which facilitates gas–liquid transport and can improve performance under gas-evolving conditions [33].

### 2.3. Electrochemical Performance and Durability Characterization

Single-cell performance and durability tests were conducted at 80 °C, with both the cell and the feed water temperature-controlled. Deionized water was supplied at 15 mL min^−1^. Measurements were carried out using a laboratory-built electrolysis station coupled to a potentiostat/galvanostat (SP-150 with VMP 3B-20, BioLogic, Seyssinet-Pariset, France).

Prior to measuring I–V, the PEMWE unit cell was activated under constant voltage that applied 1.55 V. I–V polarization curves for performance evaluation were recorded over 1.35–2.00 V in 0.05 V per step increments. All electrochemical measurements were performed using a PEMWE single-cell setup controlled by a BioLogic potentiostat. In this configuration, all reported potentials are referenced to the standard hydrogen electrode (SHE). No external reference electrode was employed.

The Ohmic overpotential can be calculated as follow equation:

$$\eta_{\mathrm{ohm}}=i\cdot (R_{\mathrm{ohm}})=i\cdot (\frac{dV}{di})$$

where *η_ohm_* is the Ohmic overpotential, *i* is the current, and *R_ohm_ =* $\frac{dV}{di}$ is the I–V slope that voltage between 1.55 and 1.85 V [34].

I–V measurements for durability analysis were obtained over a total AST duration of 192 h, during which five I–V curves were recorded. The test sequence consisted of four accelerated stress test-2 (AST-2) blocks, and one I–V curve was measured after the completion of each block. I–V curve was collected over the range of 0.0–2.0 A cm^−2^.

**Figure 2:**
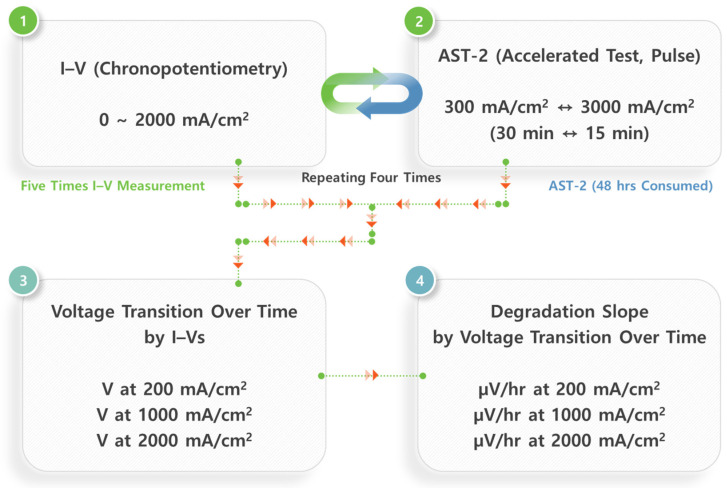
Processes for in situ durability evaluation of CL in PEMWE (The workflow proceeds from ① I–V measurement → ② AST-2 pulse test, which is alternately repeated, and then advances to ③ voltage tracking at fixed current densities → ④ calculation of degradation slopes (µV h^−1^)).

The AST-2 protocol employed alternating current loads of 0.3 A cm^−2^ for 15 min and 3.0 A cm^−2^ for 15 min, continuously applied throughout the 192 h test. The degradation slope was quantified by tracking the change in voltage at the selected current densities across the five I–V curves and plotting the voltage evolution as a function of time. Each I–V curve was collected over the range of 0.0–2.0 A cm^−2^ to extract the cell voltages at 0.0, 1.0, and 2.0 A cm^−2^. The rate of voltage increase (µV h^−1^) was then determined using linear fitting, enabling a quantitative assessment of performance degradation under dynamic load cycling. The overall sequence and timing used for the durability evaluation are shown in Figure 2. Post-test characterization focused on transmission electron microscopy (TEM) to evaluate catalyst particle size distributions before and after AST.

## 3. Results and Discussion

### 3.1. Properties of Ionomer Structure and Dispersion

The five PFSIs were classified by side-chain architecture (LSC, MSC, SSC) and equivalent weight (EW), as depicted in Figure 1 and summarized in Table 1. EW reflects the relative contribution of the side-chain molecular weight and the number of tetrafluoroethylene (TFE) repeat units in the backbone.

**Figure 3:**
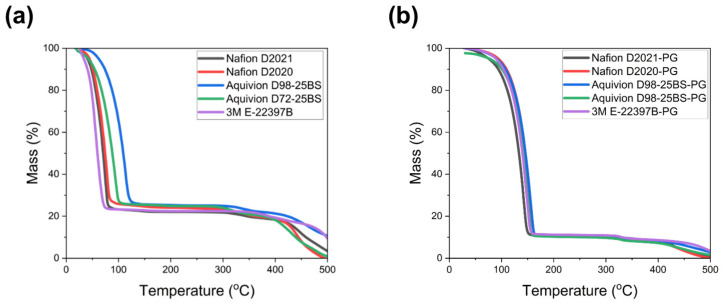
Thermogravimetric analysis (TGA) of ionomer dispersions. (**a**) As-received (W/A) dispersions showing nominal solids (20 or 25 wt%) and polymer degradation onset > 300–400 °C. (**b**) PG-substituted dispersions confirming 10 wt% solids and complete removal of water/alcohol (no mass loss at 50–150 °C).

To isolate solvent effects from ionomer chemistry, we compared as-received dispersions (water or water/alcohol, W/A) with their PG-substituted counterparts. Thermogravimetric analysis (Figure 3) verified that the as-received dispersions contained their nominal solid contents (20 wt% for Nafion/3M and 25 wt% for Aquivion), while all PG-substituted dispersions exhibited a consistent 10 wt% solid content. For the PG-based samples, no mass loss was detected in the 50–150 °C region, confirming that water and alcohol were completely removed during the solvent-substitution process and that PG remained as the sole residual solvent. Polymer degradation appeared above ~300–400 °C, as expected.

**Figure 4:**
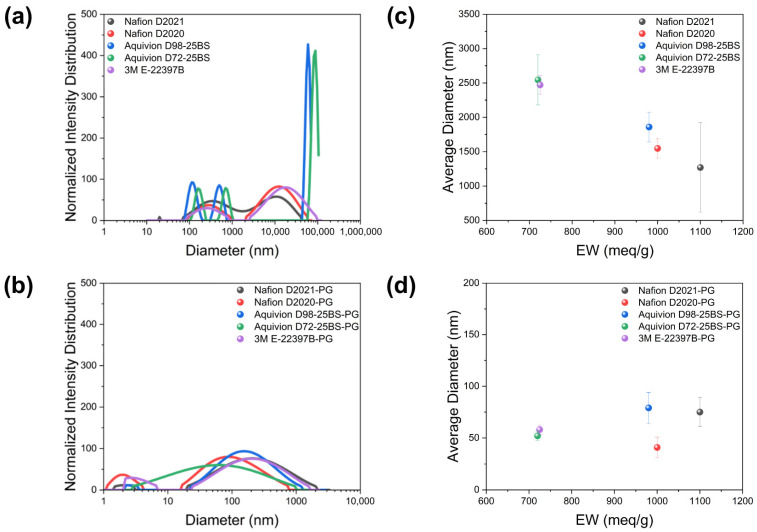
DLS of ionomer dispersions, measured five times. (**a**,**b**) As-received (W/A): bi/tri-modal, >1 μm averages with EW-dependent increase at lower EW. (**c**,**d**) PG-substituted: sub-100 nm averages with EW dependence largely suppressed.

Dynamic light scattering (DLS) revealed the core dispersion trend (Figure 4 and Table 2). For as-received (W/A) dispersions, large aggregates with bi-/tri-modal distributions and average diameters > 1 μm; within this regime, lower EW correlates with larger aggregates. After PG substitution, distributions collapse to sub-100 nm sizes with uni-/narrow bi-modal features; the EW dependence largely disappears within experimental uncertainty. Furthermore, the PG-based ionomer dispersions exhibited small hydrodynamic diameters below 100 nm in DLS measurements, indicating that water or water/alcohol components were effectively and completely removed during the solvent-substitution process.

These results show that PG strongly improves ionomer-solvent affinity, suppressing hydrophobic-backbone driven aggregation and decoupling dispersion size from EW/side-chain chemistry at the “ionomer-only” level. This sets a controlled baseline to examine how ionomer structure, rather than solvent choice, governs ink/CL properties and electrochemical behavior.

### 3.2. Structure of Catalyst Ink and Wettability of CL

**Figure 5:**
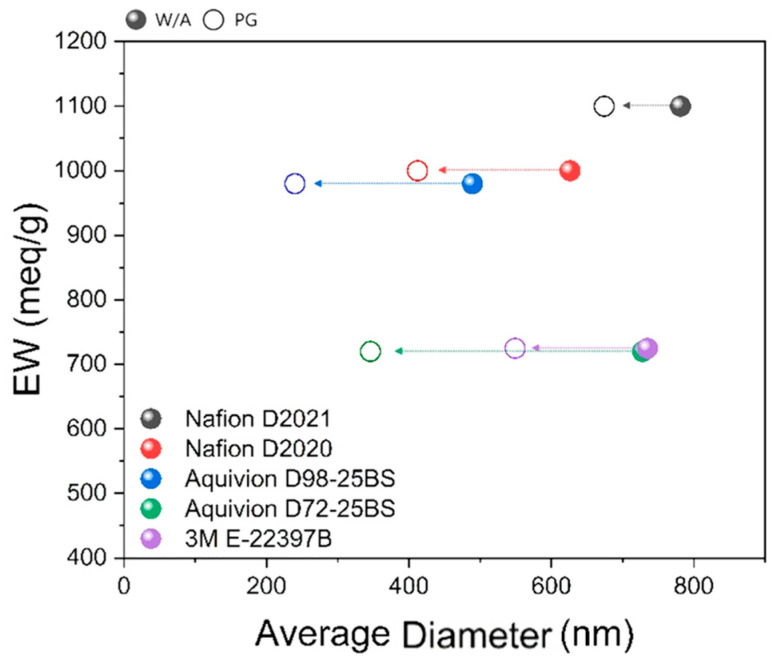
Average diameter of catalyst-ionomer agglomerates in inks: comparison between W/A (filled symbols) and PG (open symbols). Arrows indicate size reduction upon PG substitution.

Catalyst inks were prepared to probe catalyst-ionomer agglomeration under conditions identical to CL fabrication. As summarized in Figure 5, all five ionomers show a leftward shift to smaller agglomerate sizes after PG substitution. Consistent with the ionomer-only DLS, PG reduces the characteristic agglomerate size by several hundred nanometers, and no monotonic dependence on EW or side-chain length remains. Thus, the dispersion environment (PG) dominates agglomeration, while chemistry-dependent differences are expected to emerge during film formation on the porous CL rather than in bulk dispersion.

**Figure 6:**
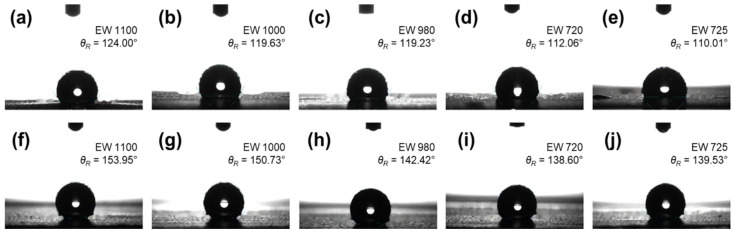
Static water contact angles (*θ_R_*) of CLs prepared with each ionomer, measured five times: (**a**–**e**) W/A-based; (**f**–**j**) PG-based.

**Figure 7:**
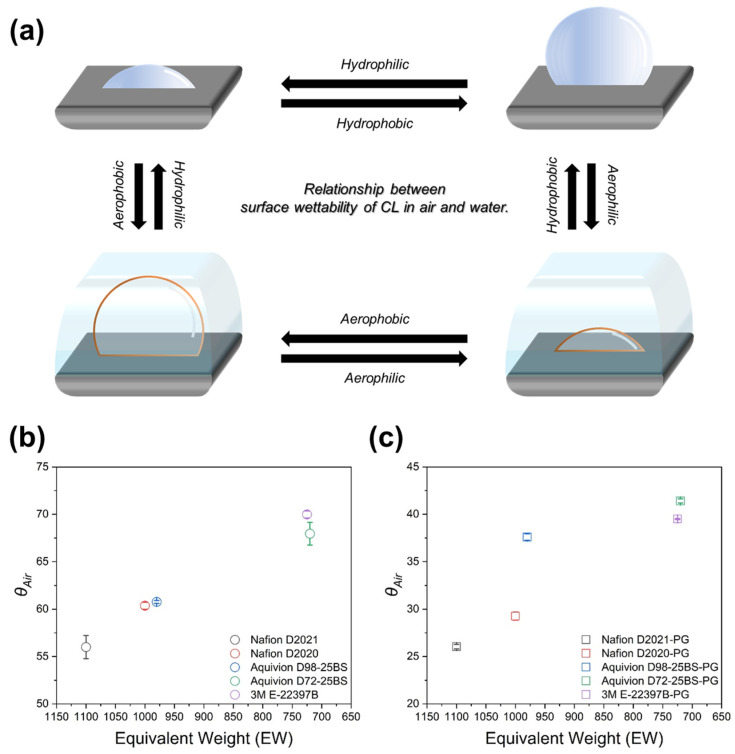
(**a**) Relationship between surface wettability of CL in air and aerophobicity in water; bubble-in-water contact angle (*θ_Air_* = 180° − *θ_R_*) vs. EW, measured five times for (**b**) W/A-based and (**c**) PG-based CLs. All data are presented as open-circle markers with error bars.

CL wettability was evaluated by *θ_R_* and the derived aerophobicity (*θ_Air_* = 180° − *θ_R_*) (Figure 6 and Figure 7). Based on the established literature, water-in-air contact angle measurements can be used to infer aerophobicity, which serves as an indirect descriptor of bubble–solid interactions in aqueous electrochemical systems. As reported previously, more hydrophilic surfaces exhibit larger bubble contact angles, reduced bubble adhesion forces, and faster bubble detachment, thereby mitigating interfacial blockage. Such aerophobic behavior has been shown to contribute to improved CL performance and durability during gas-evolving reactions by facilitating efficient gas removal and maintaining active interfacial areas [35,36]. Across all ionomers, PG substitution increased *θ_R_* and therefore decreased *θ_Air_* relative to W/A; i.e., PG renders the CL surface less aerophobic. Because bubble management in gas-evolving CLs (HER/OER) depends on a balance between water supply and bubble release, subsequent transport improvements are unlikely to arise from aerophobicity alone; PG-enabled microstructural uniformity and reduced ohmic/transport resistances are also implicated.

Numerically, *θ_R_* in the W/A set was 124.00°, 119.63°, 119.23°, 112.06°, and 110.01° for Nafion D2021, Nafion D2020, Aquivion D98-25BS, Aquivion D72-25BS, and 3M E-22397B, respectively, whereas the PG set showed 153.95°, 150.73°, 142.42°, 138.60°, and 139.53° in the same order (Figure 6). For both solvent sets, *θ_Air_* increased approximately linearly with decreasing EW (Figure 7), consistent with the higher sulfonic acid group density at lower EW. In other words, the lower-EW ionomers exhibit a higher density of sulfonic acid groups, which promotes the formation of more abundant and better-connected hydrophilic domains within the CL. These enlarged ionic pathways enhance proton transport and improve water accessibility during operation. The increased hydrophilic domain distribution also induces a more aerophobic surface character, facilitating efficient gas removal from the CL. This structural behavior is consistent with the contact-angle measurements, which show that lower-EW ionomers yield more hydrophilic surfaces. Combined with the sub-100 nm ionomer aggregates observed by DLS in PG, these results indicate that ionomer structure (EW/side-chain), not solvent variability, dictates CL wettability and the downstream electrochemical behavior.

### 3.3. Effect of the Ionomer Structure on the Performance of CL

**Figure 8:**
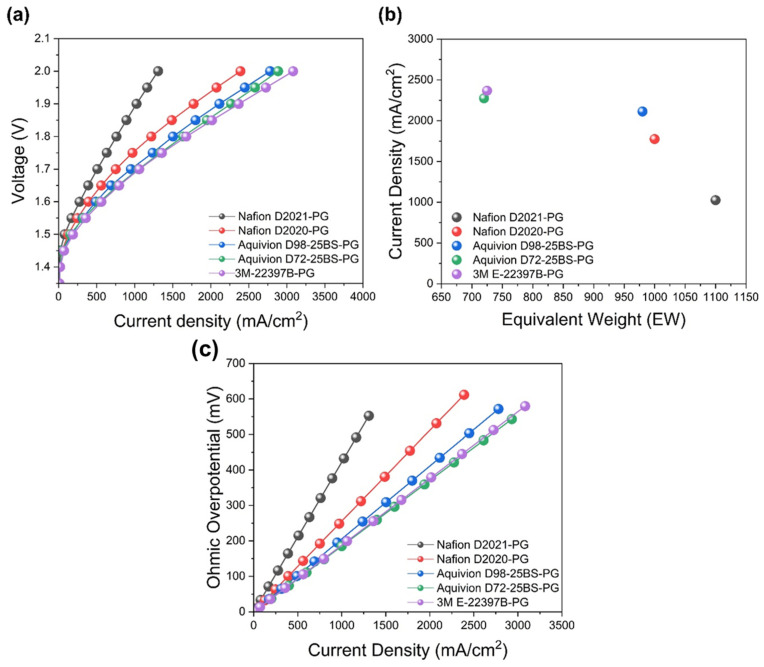
Performance of PG-based HER-CLs: (**a**) I–V curves; (**b**) current density at 1.9 V vs. EW; (**c**) ohmic overpotential vs. current density extracted from I–V curves.

To remove solvent bias, HER-CLs were fabricated only with PG-based ionomer dispersions. Accordingly, the current density was evaluated at a cell voltage of 1.9 V, which represents a practically relevant operating condition for PEMWE. At this voltage, differences in CL structure, interfacial properties, and bubble removal behavior are sensitively reflected in the measured current density [37,38]. The resulting I–V curves (Figure 8a) and the current densities at 1.9 V (Table 3) show a clear monotonic trend with EW: current density increases as EW decreases (Nafion D2021-PG 1025 < Nafion D2020-PG 1775 < Aquivion D98-25BS-PG 2113 < Aquivion D72-25BS-PG 2275 < 3M E-22397B-PG 2367 mA cm^−2^ from Figure 8b). J.-H. Park et al. has reported that SSC/MSC ionomers (Aquivion, 3M) formed thicker self-assembled ionomer films (>140 nm) on SiO_2_/Si wafer than LSC Nafion. It was experimentally confirmed that thicker films improved water retention at the HER-CL, sustaining proton conduction under gas-evolving, locally drier conditions [25]. The ohmic overpotential extracted from I–V curves is highest for LSC Nafion and lower for SSC/MSC ionomers (Figure 8c), consistent with better hydration and percolation of the proton-conducting phase.

Wettability complements these effects. Within the PG set, lower-EW ionomers exhibit larger *θ_Air_* (Figure 7c), favoring bubble detachment and reducing interfacial blockage. Overall, the performance hierarchy with PG reflects a convolution of thicker ionomer films (hydration/transport) and favorable surface energetics (bubble release) rather than differences originating from ink-state agglomeration (which showed no systematic EW dependence in PG).

### 3.4. Effect of the Ionomer Structure on the Durability of CL

**Figure 9:**
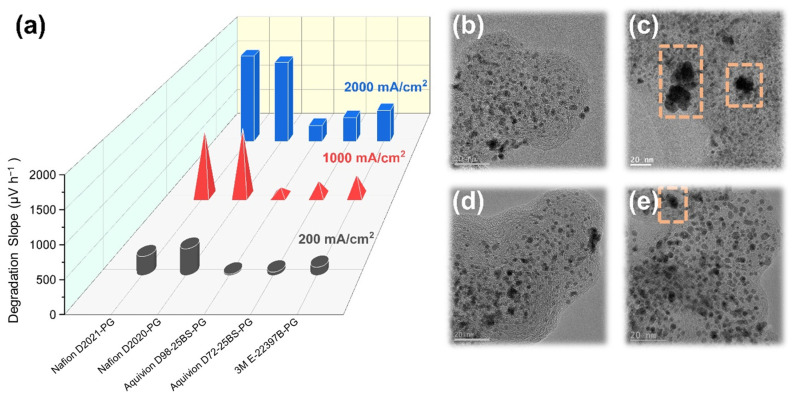
Durability of PG-based HER-CLs: (**a**) degradation slopes (Δ*V*/Δ*t*) at 200, 1000, and 2000 mA cm^−2^; (**b**–**e**) TEM images of Pt/C before and after AST (representative, Nafion D2020-PG vs. Aquivion D98-25BS-PG). The dashed pink boxes indicate regions showing pronounced Pt degradation, such as particle agglomeration and local loss of contrast, highlighting areas where severe catalyst deterioration occurred.

Durability was evaluated under the AST-2 protocol (0.3 A cm^−2^ for 30 min/3.0 A cm^−2^ for 15 min, repeated four times at 48 h intervals). Degradation slopes (Δ*V*/Δ*t*) were computed from I–V potentials at 200, 1000, and 2000 mA cm^−2^. Results split into two groups (Figure 9a and Table 3). LSC Nafion (D2021-PG, D2020-PG) shows large slopes across all currents (e.g., ~1500–1670 μV h^−1^ at 2000 mA cm^−2^), whereas SSC/MSC (Aquivion D98-25BS-PG, D72-25BS-PG, and 3M E-22397B-PG) exhibits substantially smaller slopes (e.g., ~314–605 μV h^−1^ at 2000 mA cm^−2^), with Aquivion D98-25BS-PG the lowest among the five dispersions.

The ionomer’s chemical structure and two experimentally supported factors rationalize the superior stability of SSC/MSC. First, PFSIs are separated in terms of ionomer structure such as number of CF_2_, CF, and ether linkages. Based on these, Nafion has the most unfavorable conditions for CF_2_ oxidation [39,40,41], H radical attack on CF groups [40], and OH radical attack on ether linkages [41], showing the highest degradation of CL in PEMWE. This is because Nafion has a greater number of CF_2_, CF, and ether linkages, making it more susceptible to oxidation and radical attack. On the other hand, the Aquivion ionomer exhibited the lowest degradation slope, which can be explained by its structure. This is because the Aquivion ionomer is less susceptible to oxidation and radical attack due to having fewer CF_2_, CF, and ether linkages compared to the Nafion ionomer. Among ionomers with various EWs, Nafion EW 1000 and Aquivion EW 980 can be specifically discussed in terms of durability because they have similar EW and the number of TFE repeating units, with the only difference being the number of CF_2_, CF, and ether linkages. Second, thicker ionomer films (SSC/MSC) correlate with lower ohmic overpotential, implying better hydration/percolation and greater mechanical/chemical shielding of Pt/C during dynamic loading, limiting mobility-driven coarsening. Third, TEM comparisons before/after AST show notable Pt growth for Nafion D2020-PG, but minimal growth for Aquivion D98-25BS-PG (Figure 9b–e), consistent with stronger ionomer-catalyst binding and/or better local hydration.

Among PG-based binders tested, Aquivion D98-25BS-PG offers the lowest degradation slope with high performance, 3M E-22397B-PG delivers the highest current density with moderate slopes, and Nafion D2021/2020-PG shows the poorest durability. Thus, SSC (EW ≈ 980) emerges as a robust durability choice for HER-CL binders under PG, with MSC as a high-performance alternative.

## 4. Conclusions

Using a single-solvent framework based on propylene glycol (PG) removed solvent-to-solvent variability and allowed the intrinsic influence of PFSI structure, i.e., side-chain architecture and equivalent weight (EW), to be isolated across the ink, CL, and cell responses. PG substitution collapsed ionomer-only aggregates from micron scales in water/alcohol to sub-100 nm for all five ionomers; the EW dependence seen in the as-received dispersions largely disappeared. In catalyst inks, agglomerate sizes were likewise reduced without a systematic trend with EW, indicating that subsequent differences originate primarily during film formation on the catalyst rather than from the ink state itself.

Within this PG baseline, CL wettability became uniformly more hydrophobic (higher *θ_R_*, thus lower *θ_Air_*) than in water/alcohol, yet a clear structural signature remained: *θ_Air_* increased with decreasing EW. This behavior is consistent with a higher sulfonic-acid group density at lower EW, which tunes surface energetics relevant to gas–liquid transport and establishes the interfacial environment encountered during hydrogen evolution.

The performance hierarchy under PG, i.e., Nafion D2021-PG < Nafion D2020-PG < Aquivion D98-25BS-PG < Aquivion D72-25BS-PG < 3M E-22397B-PG, reflects two PG-enabled material effects. Short and mid-side-chain ionomers form thicker ionomer films on the catalyst (≥~140 nm), which enhance water retention and percolation of proton-conducting domains, and they exhibit lower ohmic overpotentials than long side-chain Nafion. As a result, the increase in current density with decreasing EW is governed by hydration-driven transport and film connectivity rather than by aerophobicity alone.

Durability under dynamic loading (AST-2) separated into two groups: long side-chain Nafion showed the largest degradation slopes at all probed currents, whereas short/mid-side-chain ionomers were markedly more stable, with Aquivion D98-25BS-PG the most durable. Transmission electron microscopy before and after cycling corroborated the electrochemical trends, revealing pronounced Pt growth for Nafion D2020-PG but minimal growth for Aquivion D98-25BS-PG, consistent with more effective ionomer-mediated protection of the catalyst. While simple counts of CF_2_/CF/ether linkages offer a qualitative rationale for chemical robustness, the principal evidence rests on the electrochemical slopes and microscopy.

For HER-CL binders, a short side-chain ionomer with EW ≈ 980 (Aquivion D98-25BS-PG) provides the most favorable durability while maintaining high performance, whereas a mid-side-chain option (3M E-22397B-PG) maximizes current density with moderate stability and long side-chain Nafion performs worst in both respects. These findings offer practical guidance for PEMWE CL design: select lower-EW SSC/MSC chemistries to promote thicker, better-hydrated ionomer networks with reduced ohmic losses, and tune surface energetics within the PG framework for balanced bubble management. Building on the solvent-replacement approach applicable to CL fabrication and the improved understanding of ionomer–solvent interactions relevant to oxygen-electrode formation, the insights gained in this study can be extended to a wide range of electrochemical systems that rely on polymeric binders. In particular, the design principles, dispersion control, and film-formation behavior elucidated here are directly transferable to electrode fabrication for PEM fuel cells (PEMFCs), PEMWE anodes, anion-exchange-membrane fuel cells (AEMFCs), and anion-exchange-membrane water electrolysis (AEMWEs).

Overall, under the PG single-solvent baseline examined in this study, the results suggest that ionomer structure has a stronger influence on CL performance and durability than solvent choice; however, this conclusion is valid only within the specific range of PG-based formulations investigated here and should not be overgeneralized beyond these conditions. Future research should therefore investigate how ionomer structure, solvent compatibility, and processing strategies can be tailored to meet the distinct chemical environments and hydration requirements of these systems. Such efforts would broaden the applicability of solvent-replacement methodologies and support the development of more robust, efficient, and durable electrode architectures across diverse electrochemical technologies.

## Figures and Tables

**Table 1:** Summary of ionomer properties: EW, estimated TFE repeat units, and side-chain architecture.

Ionomer Dispersion	EW (g eq^−1^)	TFE (m)	x (CF_2_-CF-CF_3_-O) and y (CF_2_)
Nafion D2021 (LSC)	1100	6.6	x = 1 and y = 2
Nafion D2020 (LSC)	1000	5.0	x = 1 and y = 2
Aquivion D98-25BS (SSC)	980	7.0	x = 0 and y = 2
Aquivion D72-25BS (SSC)	720	4.4	x = 0 and y = 2
3M E-22397B (MSC)	725	3.5	x = 0 and y = 4

**Table 2:** Average particle size of ionomer dispersions (mean ± SD) before and after PG substitution.

Ionomer Dispersion		Average Particle Size (nm)
Commercially available	Nafion D2021	1269 ± 650.9
	Nafion D2020	1547 ± 142.0
	Aquivion D98-25BS	1859 ± 217.4
	Aquivion D72-25BS	2545 ± 366.1
	3M E-22397B	2471 ± 134.6
Solvent-substituted	Nafion D2021-PG	75 ± 14
	Nafion D2020-PG	41 ± 9.9
	Aquivion D98-25BS-PG	79 ± 15
	Aquivion D72-25BS-PG	52 ± 4.7
	3M E-22397B-PG	58 ± 4.5

**Table 3:** Current density at 1.9 V and degradation slopes (μV h^−1^) at 200/1000/2000 mA cm^−2^ for PG-based HER-CLs.

Solvent-Substituted Ionomer Dispersion	Current Density (mA/cm^2^ at 1.9 V)	Degradation Slope (μV/h) at Three Applied Current Densities (mA/cm^2^)		
		200	1000	2000
Nafion D2021-PG	1025	290	1160	1670
Nafion D2020-PG	1775	407	1180	1540
Aquivion D98-25BS-PG	2113	21.6	148	314
Aquivion D72-25BS-PG	2275	53.0	257	468
3M E-22397B-PG	2367	127	370	605

## Data Availability

The raw data supporting the conclusions of this article will be made available by the author on request.
